# Hypothalamic Sirt1 Regulates Food Intake in a Rodent Model System

**DOI:** 10.1371/journal.pone.0008322

**Published:** 2009-12-15

**Authors:** Işin Çakir, Mario Perello, Omar Lansari, Norma J. Messier, Charles A. Vaslet, Eduardo A. Nillni

**Affiliations:** 1 Division of Endocrinology, Department of Medicine, The Warren Alpert Medical School of Brown University/Rhode Island Hospital, Providence, Rhode Island, United States of America; 2 Department of Molecular Biology, Cell Biology and Biochemistry, Brown University, Providence, Rhode Island, United States of America; 3 Department of Pathology and Laboratory Medicine, Brown University, Providence, Rhode Island, United States of America; University of Hong Kong, China

## Abstract

Sirt1 is an evolutionarily conserved NAD^+^ dependent deacetylase involved in a wide range of processes including cellular differentiation, apoptosis, as well as metabolism, and aging. In this study, we investigated the role of hypothalamic Sirt1 in energy balance. Pharmacological inhibition or siRNA mediated knock down of hypothalamic Sirt1 showed to decrease food intake and body weight gain. Central administration of a specific melanocortin antagonist, SHU9119, reversed the anorectic effect of hypothalamic Sirt1 inhibition, suggesting that Sirt1 regulates food intake through the central melanocortin signaling. We also showed that fasting increases hypothalamic Sirt1 expression and decreases FoxO1 (Forkhead transcription factor) acetylation suggesting that Sirt1 regulates the central melanocortin system in a FoxO1 dependent manner. In addition, hypothalamic Sirt1 showed to regulate S6K signaling such that inhibition of the fasting induced Sirt1 activity results in up-regulation of the S6K pathway. Thus, this is the first study providing a novel role for the hypothalamic Sirt1 in the regulation of food intake and body weight. Given the role of Sirt1 in several peripheral tissues and hypothalamus, potential therapies centered on Sirt1 regulation might provide promising therapies in the treatment of metabolic diseases including obesity.

## Introduction

Sirt1 is an evolutionarily conserved NAD^+^-dependent deacetylase involved in a wide range of metabolic processes in the periphery [1]. In the liver, Sirt1 deacetylates and activates the transcriptional co-activator PGC1-alpha and the transcription factor FoxO1 to promote gluconeogenesis [2], [3]. In the adipose tissue, Sirt1 triggers fat mobilization by inhibiting peroxisome proliferator-activated receptor (PPAR-gamma), and in the pancreas repression of the uncoupling protein 2 (UCP2) by Sirt1 increases insulin secretion [1]. Sirt1 expression as well as its activity is nutrient sensitive. For example, in the liver, the Sirt1 protein level increases upon fasting [3] and decreases by high fat diet [4]. Sirt1 action in the central nervous system has been studied as well. Sirt1 has neuroprotective effects. For example, increased nuclear NAD biosynthesis and Sirt1 activation are linked to axonal protection [5], and hippocampus over expression of Sirt1 provides protection against neurodegeneration in a mouse model of Alzheimer's disease [6]. Sirt1 showed to be important in the regulation of stem cells to generate either astroglial lineage or the neuronal lineage [7]. At a global scale Sirt1 target genes in the mouse brain are deregulated by aging. This situation (at the gene expression level) can be reversed by specific over expression of central Sirt1 [8]. Brain Sirt1 expression increases by caloric restriction [9], and fasting was shown to increase brain Sirt1 protein content specifically in the hypothalamus [10]. A better understanding of hypothalamic Sirt1 action is of crucial importance since the hypothalamus is the primary brain center that interprets adiposity or nutrient related inputs to regulate energy homeostasis. Specifically, the anorexigenic proopiomelanocortin (POMC) neurons and the orexigenic NPY/AgRP neurons in the arcuate nucleus (ARC) of the hypothalamus are the major regulators of feeding and energy expenditure [11]. Although the Sirt1 expression in POMC neurons has been reported [10], its functional role in the hypothalamus has not been determined. Hypothalamic control of food intake and body weight involves the action of metabolic sensors including the mammalian target of rapamycin (mTOR) [12] and AMP activated kinase (AMPK) [13]. Because of its dependence on NAD^+^, Sirt1 also functions as a metabolic sensor [14]. Therefore, we hypothesized that hypothalamic Sirt1 represents another metabolic factor controlling food intake. Using the rat as a physiological *in vivo* model we found that ablation of Sirt1 activity or knocking-down Sirt1 gene expression at the hypothalamic level resulted in decreased food intake and body weight gain. Blocking the melanocortin receptors with the melanocortin antagonist SHU9119 attenuated the anorectic effect of Sirt1 inhibition. Fasting increased hypothalamic NAD^+^ levels and Sirt1 protein content. Inhibition of hypothalamic Sirt1 activity reversed the fasting induced decrease of FoxO1 acetylation, and resulted in increased POMC and decreased AgRP expressions. Regulation of POMC by Sirt1 depends on FoxO1 as shown utilizing in vivo and in vitro approaches. We also present data showing that hypothalamic Sirt1 regulates S6K signaling. Thus, targeting central Sirt1 signaling may show promise for the treatment of obesity and the associated disorders.

## Results

### Hypothalamic Sirt1 Regulates Food Intake and Body Weight

**Figure 1:**
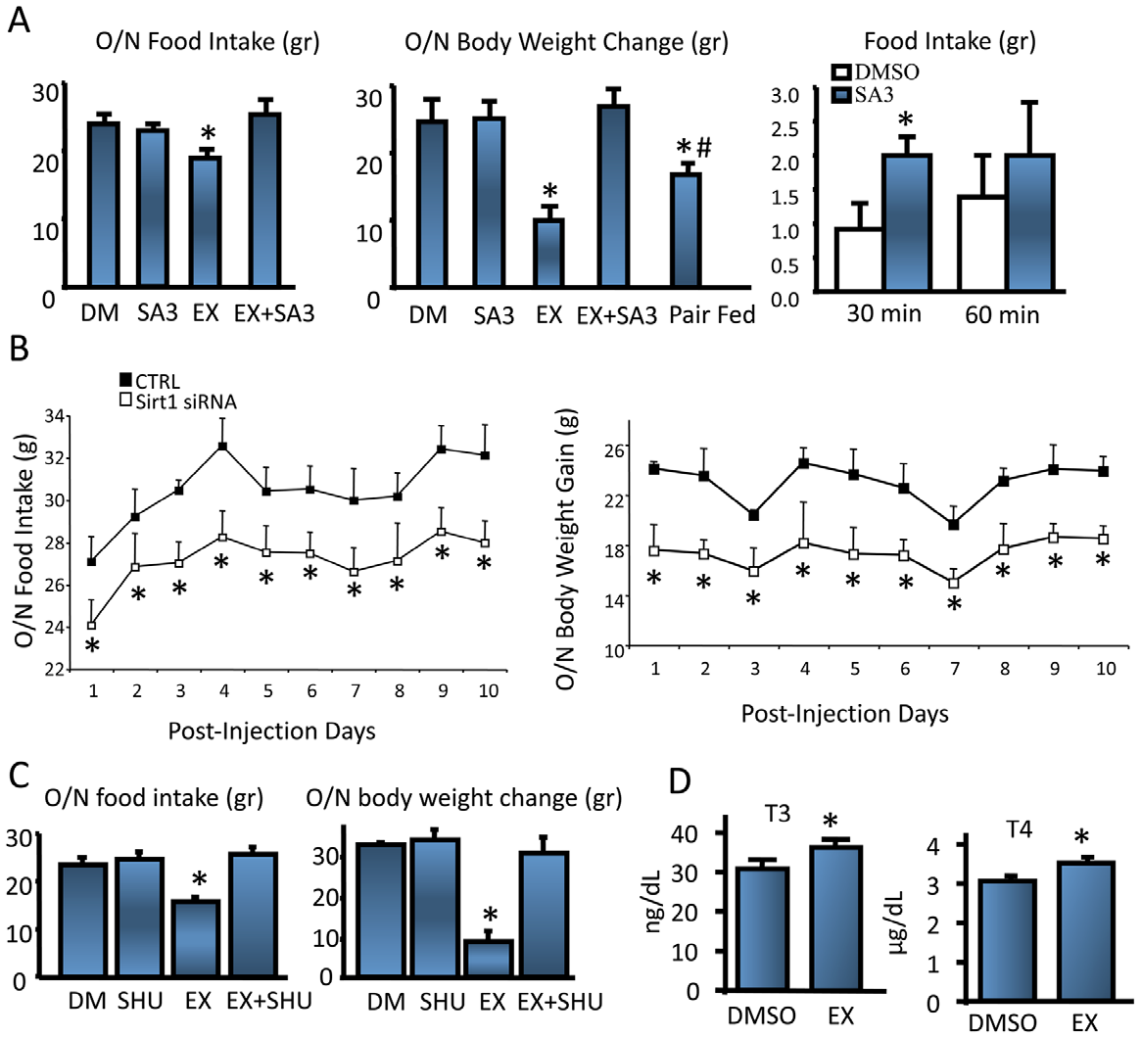
Hypothalamic Sirt1 regulates food intake through melanocortin receptors . *(A)* Pharmacological inhibition of Sirt1 decreases food intake (left) and body weight gain (middle) in fasted rats, whereas activation of Sirt1 increases food intake (right) in re-fed rats. n = 8–9, per treatment, and per group. (*B)* Food intake (left) and body weight gain (right) decreases by knocking down Sirt1 expression specifically in the ARC. n = 6–7, per treatment. (*C)* (Left) Effect of co-administration of EX527 and SHU9119 on food intake. Animals were treated the same way as in (*A)*. Food intake was measured overnight for a period of 16 hours. (Right) Effect of co-administration of EX527 and SHU9119 on body weight change. Same animals were weighed one hour before dark cycle, and in the morning. n = 5 per treatment. (*D)* Fasted rats were icv infused with DMSO (vehicle) or EX-527 as described in (*A)*. One hour after second icv infusion (49-hour of fasting) rats were sacrificed. Blood was collected at the time of decapitation. Plasma T3 and T4 was determined by RIA. (DM, DMSO; EX, EX-527; SHU, SHU9119; SA3, Sirt1 Activator 3). Values are the mean±SEM. *, 0.01<p<0.05 throughout the text (unless p is stated), vs. DMSO (A, C, D) or CTRL (B) groups. #, 0.01<p<0.05 vs. EX527 group.

To study the potential role of hypothalamic Sirt1 in energy balance we first determined its expression in the rat hypothalamus. Immunohistochemical (IHC) staining revealed a nuclear Sirt1 expression in certain hypothalamic centers involved in energy homeostasis, namely the ARC and paraventricular nucleus (PVN) of the hypothalamus (Figure S1A). By immunoblotting analysis, we also confirmed the presence of Sirt1 in the ARC, PVN, primary neurons, and in the mouse corticotropic AtT20 cell line (Figure S1B) (see below). As recently described for the mouse brain [10], Sirt1 expression is present in other brain areas such as the hippocampal formation and cortex (Figure S1C). We next wanted to determine if modulating Sirt1 activity or its expression would effect food intake and body weight of adult rats. To block Sirt1 enzymatic activity we used a potent Sirt1 inhibitor [15]. This compound (EX-527) is a small, selective inhibitor of SIRT1 that does not inhibit histone deacetylases and is specific for Sirt1 over other sirtuin family members [16], [17]. Intracerebro-ventricular (ICV) administration of EX-527 for 24 (Figure S1D, left and middle panels) or 48 hours (Figure 1A left and middle panels) in fasted rats resulted in a decreased in food intake accompanied by a reduced body weight gain. Both the food intake and body weight changes induced by EX527 were Sirt1 specific because both parameters were reversed by co-administration of a Sirt1 activator [18] (Sirt1 activator 3, or SA3) at a dose, which alone did not change either food intake or weight gain (Figure 1A). Induction of hypothalamic Sirt1 activity by ICV infusion of SA3 to re-fed rats (Figure S1D, right panel) resulted in increased short-term food intake (Figure 1A, right). To confirm the specificity of pharmacological Sirt1 inhibition, and to observe whether the acute manipulation of Sirt1 activity could be recapitulated in the long-term, we knocked down Sirt1 expression by infusing Sirt1 specific siRNAs directly into the ARC (Figure S1F). Consequently, Sirt1 expression was knocked-down 40% at the protein level (Figure S1E), which was confirmed by the decreased number of Sirt1 positive ARC nuclei (Figure S1F, G). Rats with diminished ARC Sirt1 protein consumed less food (Figure 1B, left), and gained less weight compared to the control group (Figure 1B, right).

### The Effect of Hypothalamic Sirt1 on Energy Balance Is Mediated through Melanocortin Receptors

One of the main regulators of energy homeostasis at the hypothalamic level is the melanocortin system. If the effect of Sirt1 on energy balance is mediated through central melanocortin signaling, blocking melanocortin receptors should reverse the anorexogenic outcome of Sirt1 inhibition. To test this hypothesis, we ICV infused rats with EX-527 and the melanocortin antagonist SHU9119. We used a dose of SHU9119 that did not change the overnight food intake or body weight (Figure 1C). Administration of the melanocortin antagonist together with the Sirt1 inhibitor completely attenuated the decreased food intake or the weight gain induced by the Sirt1 inhibitor alone (Figure 1C). This result also shows that the effect of the Sirt1 inhibitor on food intake was specific and was not due to nonspecific effects such as food aversion. It has long been accepted that the thyroid hormone is an important determinant of overall energy consumption in humans and animals by affects uncoupling proteins (UCP-1, UCP-2, and UCP-3) in brown adipose tissue (BAT), muscle, and other tissues in combination with an activation of the sympathetic nervous system to accelerate ATP turnover [19]. The TRH peptide produced in neurons of the PVN is released from the median eminence to stimulate the biosynthesis and secretion of thyroid stimulating hormone (TSH) from the pituitary, which in turn, stimulates the biosynthesis of thyroid hormone, thyroxine (T_4_), and it modified product triidothyronine (T_3_) [22]. We had previously showed that the α-MSH peptide derived from POMC processing is one of the main regulators of the hypothalamic-pituitary-thyroid (HPT) axis [21], [20] by up-regulating the TRH neuron in the PVN. Since the inhibition of hypothalamic Sirt1 induced an anorectic effect in a SHU9119 dependent manner, we measured the T_3_ and T_4_ plasma levels of EX527 infused animals. As shown in Figure 1D, inhibition of hypothalamic Sirt1 resulted in a significant rise in plasma T_3_ and T_4_ levels, strongly suggesting that the melanocortin system was positively activated when Sirt1 activity was inhibited.

### Sirt1 Regulates Melanocortin System at the Level of ARC

**Figure 2:**
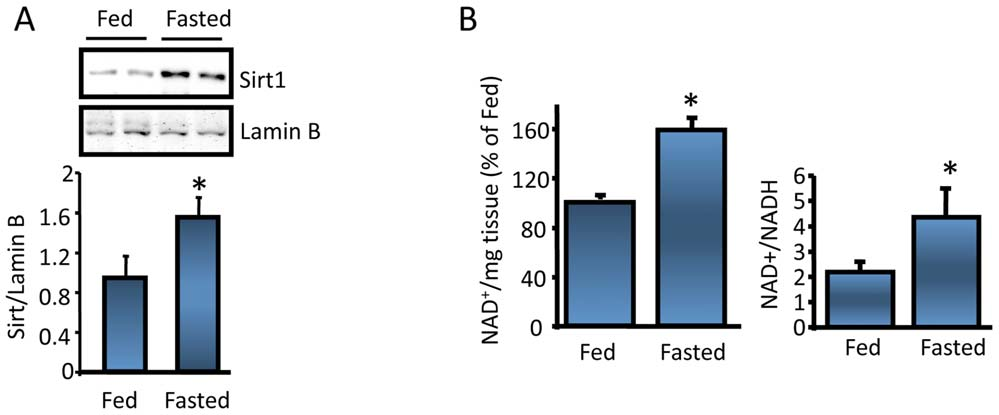
Hypothalamic Sirt1 expression and activity are nutrient sensitive. *(A*) 24 hr fasting increases hypothalamic nuclear Sirt1 protein levels. n = 4. (*B*) Fasting increases NAD+ levels as well as the NAD+/NADH ratio in the ARC. Of note, POMC-derived peptide α-MSH level decreases by fasting in the ARC (Figure S2C). The experiment was done twice using 3 rats per group. Values are the mean±SEM. *, vs. fed.

**Figure 3:**
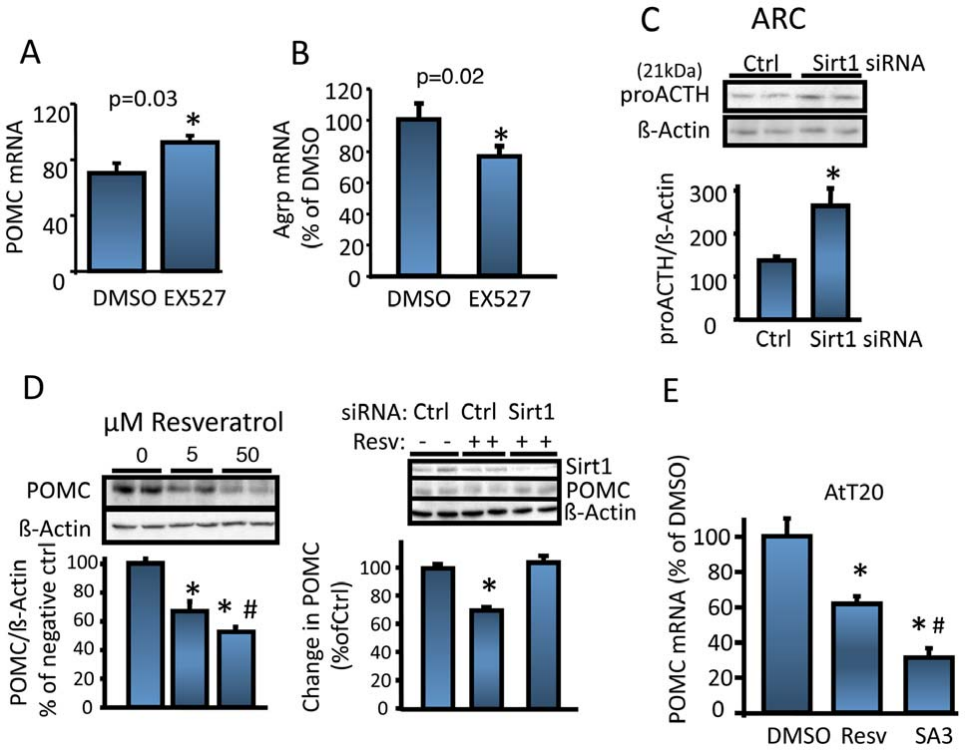
Sirt1 regulates central melanocortin system. (*A)* Inhibition of hypothalamic Sirt1 increases ARC POMC expression of fasted rats. n = 5. (*B*) ARC Agrp expression of animals in (*A*). n = 5. (*C)* ProACTH increases upon Sirt1 knock-down in the ARC. Intra-ARC siRNA infused rats were decapitated at postinjection day 2, and ARCs were analyzed by western blotting using an antibody that recognized POMC, as well as proACTH (21 kDa). Bottom graph shows the quantification of the band intensities. Values are normalized to β-actin. n = 5. (*D*) Resveratrol decreases POMC expression in AtT20 cells in a Sirt1- and dose-dependent manner. The experiments were done twice with n = 3 per group. #, p<0.05 vs. 5 µM resveratrol group. (Resv =  resveratrol). (*E*) Resveratrol (50 µM) or SA3 (50 µM) treatment of AtT20 cells decreases POMC mRNA levels detected by real time RT-PCR. The experiment is done twice, in triplicate. #, p<0.05 vs. resveratrol group. *, vs. DMSO or Ctrl groups.

It has been recently shown in mice that hypothalamic Sirt1 protein levels are increased by fasting [10]. We confirmed this finding in the rat hypothalamic nuclear extracts (Figure 2A). The fasting induced increase in hypothalamic Sirt1 protein level was probably due to post-transcriptional regulations, because Sirt1 mRNA level was unaltered by fasting (data not shown). In addition, as depicted in Figure 2B fasting increased ARC NAD^+^ levels and the NAD^+^/NADH ratio. Higher NAD^+^ levels, in addition to an elevated hypothalamic Sirt1 protein expression, suggest that Sirt1 deacetylase activity increases upon fasting. POMC (Figure S2A) and AgRP neurons together with the melanocortin receptors constitute the hypothalamic part of the central melanocortin system [23] important in the regulation of food intake and energy expenditure. Upon fasting POMC gene expression decreases whereas AgRP increases. Figure S2B shows that Sirt1 is expressed virtually in all POMC neurons in the ARC, in agreement with a previous report [10]. Since hypothalamic Sirt1 regulates food intake through melanocortin receptors (Figure 1C), we investigated the effect of inhibition of hypothalamic Sirt1 in POMC and AgRP expression. ICV infusion of EX527 to fasted rats resulted in an increased POMC (Figure 3A) and a decreased AgRP mRNA levels (Figure 3B). We next wanted to determine if the changes seen in POMC mRNA were also reflected at the POMC processing products level. With the antibodies we used (see experimental procedures), the ∼31 kDa POMC precursor normally present in the ARC was undetectable by the western blot analysis probably due to a fast processing and low protein content of this precursor [24], (see Figure S3A), a phenomenon commonly seen for proneuropeptide precursors. However, we detected the 21 kDa proACTH intermediate form (Figure 3C) that showed to increase when the ARC Sirt1 expression was knocked-down by siRNA infusion (Figure S1E). In support of these in vivo studies, we also analyzed the effect of Sirt1 expression on POMC in primary hypothalamic cultures obtained from E17.5 rat embryo diencephalons [21], [25]. These cultures contain POMC neurons, and secrete α-MSH into the media. When the Sirt1 expression was knocked down by a Sirt1 shRNA adenovirus (Figure S3B), the α-MSH peptide in the culture media increased (Figure S3C) as analyzed by a specific RIA.

### Sirt1 Regulates POMC Expression In Vitro

To study the regulation of POMC expression by Sirt1, we used the AtT20 cell line that has been shown to be a useful *in vitro* model for this type of studies [26], [27]. AtT20 cells express Sirt1 (Figure S1B), POMC, and several key transcription factors involved in POMC regulation such as Stat3 and FoxO1 (data not shown). The corticotropic AtT20 cells process POMC to generate ACTH, but does not generate α-MSH because they lack the prohormone convertase 2 (PC2) essential for the conversion of ACTH to α-MSH [20] (Figure S3A). Treatment of AtT20 cells with the Sirt1 activating compound resveratrol resulted in decreased POMC protein levels (Figure 3D, left) and decreased ACTH secreted into the media (Figure S3D). The effect of resveratrol was Sirt1 specific because the siRNA mediated knock-down of Sirt1 blunted the effect of resveratrol on POMC (Figure 3D, right). In addition, SA3 induced a more potent decrease in ACTH secretion (Figure S3D). Finally, stimulation of AtT20 cells with resveratrol or SA3 decreased the POMC mRNA levels (Figure 3E) in a similar fashion to that observed in ACTH secretion. Altogether, the *in vivo* combined with the *in vitro* results strongly supports an inhibitory role for Sirt1 on POMC gene regulation in a pre-translational manner.

### Forkhead Transcription Factor FoxO1 Mediates Sirt1's Effect on POMC

**Figure 4:**
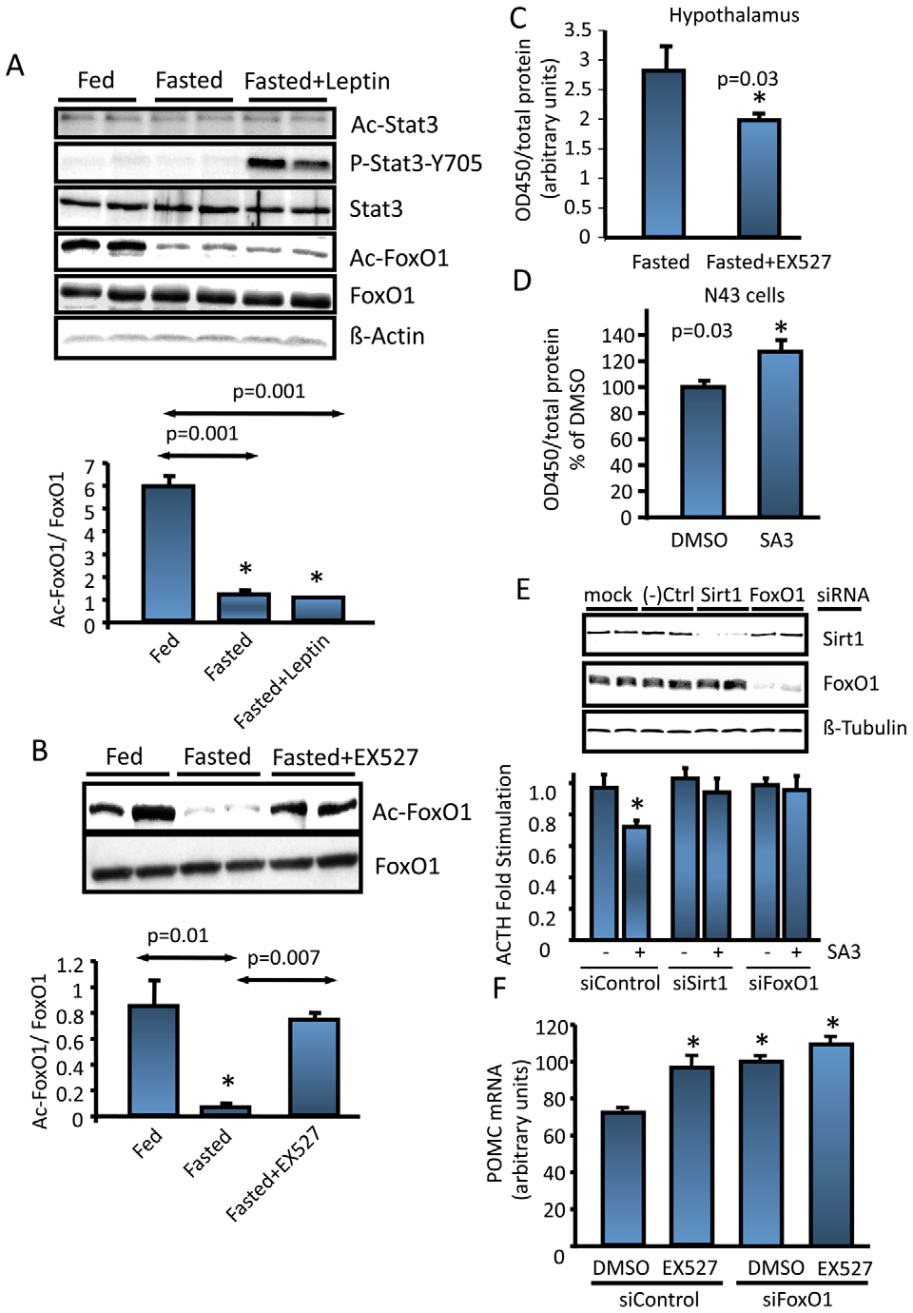
FoxO1 deacetylation couples Sirt1 action to POMC expression. *(A)* Representative western blots showing the acetylation status of FoxO1 and Stat3 upon fasting and intraperitoneal leptin administration. 5 animals per group were evaluated. (*B)* Fasting induced FoxO1 deacetylation is Sirt1 dependent. Rats were icv treated with DMSO (fed and fasted groups) or EX-527 as in Figure 1D. ARCs were analyzed for the expression of Ac-FoxO1 and total FoxO1 by western blotting. 5 animals per group were evaluated. (*C)* Inhibition of Sirt1 activity decreases FoxO1 activity *in vivo*. (*D)* Activation of Sirt1 in hypothalamic N43 cell line increases FoxO1 activity. (*E)* FoxO1 knock-down abrogates the inhibitory effect of Sirt1 on POMC expression. AtT20 cells were transfected with the corresponding siRNAs as described in Figure 3D. 24 hr after transfection the cells were serum starved and treated either with DMSO or SA3 (5 µM) for 6 hours. ACTH in the media was measured by RIA. (*F*) Hypothalamic knock-down of FoxO1 reverses the affect of Sirt1 inhibition on POMC (*, vs. siControl DMSO group). Values are the mean±SEM. *, vs. respective control groups unless stated otherwise.

The transcription factors Stat3 and FoxO1 (Figure S4A) are involved in the regulation of POMC gene expression, and both were shown to functionally interact with Sirt1 [28], [29]. Their acetylation states contribute to their function and/or DNA binding [29], [30]. Therefore we analyzed whether FoxO1 or Stat3 acetylation was nutrient sensitive in the ARC. Figure 4A shows that whereas Stat3 acetylation did not change upon fasting, there was a significant decrease in FoxO1 acetylation. Under our experimental conditions, leptin (intraperitoneally) did not change the acetylation state of either protein, suggesting that the fasting induced decrease in FoxO1 acetylation was leptin-independent. Of note, leptin did not change the ARC Sirt1 protein level (data not shown). Blocking hypothalamic Sirt1 activity (Figure S4B) reversed the decrease in FoxO1 acetylation, suggesting that fasting induced deacetylation of FoxO1 required Sirt1 (Figure 4B). Fasting induced decrease in p53 acetylation [10] was also reversed by inhibition of Sirt1 function, and Stat3 acetylation was insensitive to Sirt1 inhibition (data not shown). We next tested whether Sirt1 alters FoxO1 activity. For this purpose we used an assay that indirectly measures the amount of FoxO1 bound to a forkhead box DNA element immobilized in a 96-well plate. Inhibition of hypothalamic Sirt1 resulted in decreased binding of FoxO1 (Figure 4C). We also treated N43 immortalized hypothalamic cell line [31] with the Sirt1 activator SA3, which was accompanied by increased FoxO1 binding (Figure 4D). Since FoxO1 suppresses POMC transcription [26], [27], we tested whether FoxO1 was necessary for the regulation of POMC by Sirt1. Therefore, we knocked-down either Sirt1 or FoxO1 in AtT20 cells followed by a treatment with SA3. As expected, ACTH secretion was decreased upon SA3 treatment of the negative control siRNA-transfected cells (Figure 4E). In the absence of SA3, siSirt1, or siFoxO1 did not lead to significant changes in POMC expression in this cell line, probably because of the basal level of POMC expression is not inhibited by FoxO1 under the culture conditions used. As shown for resveratrol in Figure 3D, the SA3-induced decrease in ACTH was Sirt1 dependent, since knock-down of Sirt1 abolished this effect (Figure 4E). Importantly, in the presence of intact Sirt1, knock-down of FoxO1 was able to reverse the inhibitory effect of SA3 on POMC (Figure 4E). Similarly, in vivo knock-down of hypothalamic FoxO1 expression (Figure S4C) reversed the EX527-induced increase in POMC expression (Figure 4F) as well as the EX527-induced decrease in the AgRP expression (data not shown).

### Hypothalamic Sirt1 Regulates S6 Kinase (S6K) Pathway

**Figure 5:**
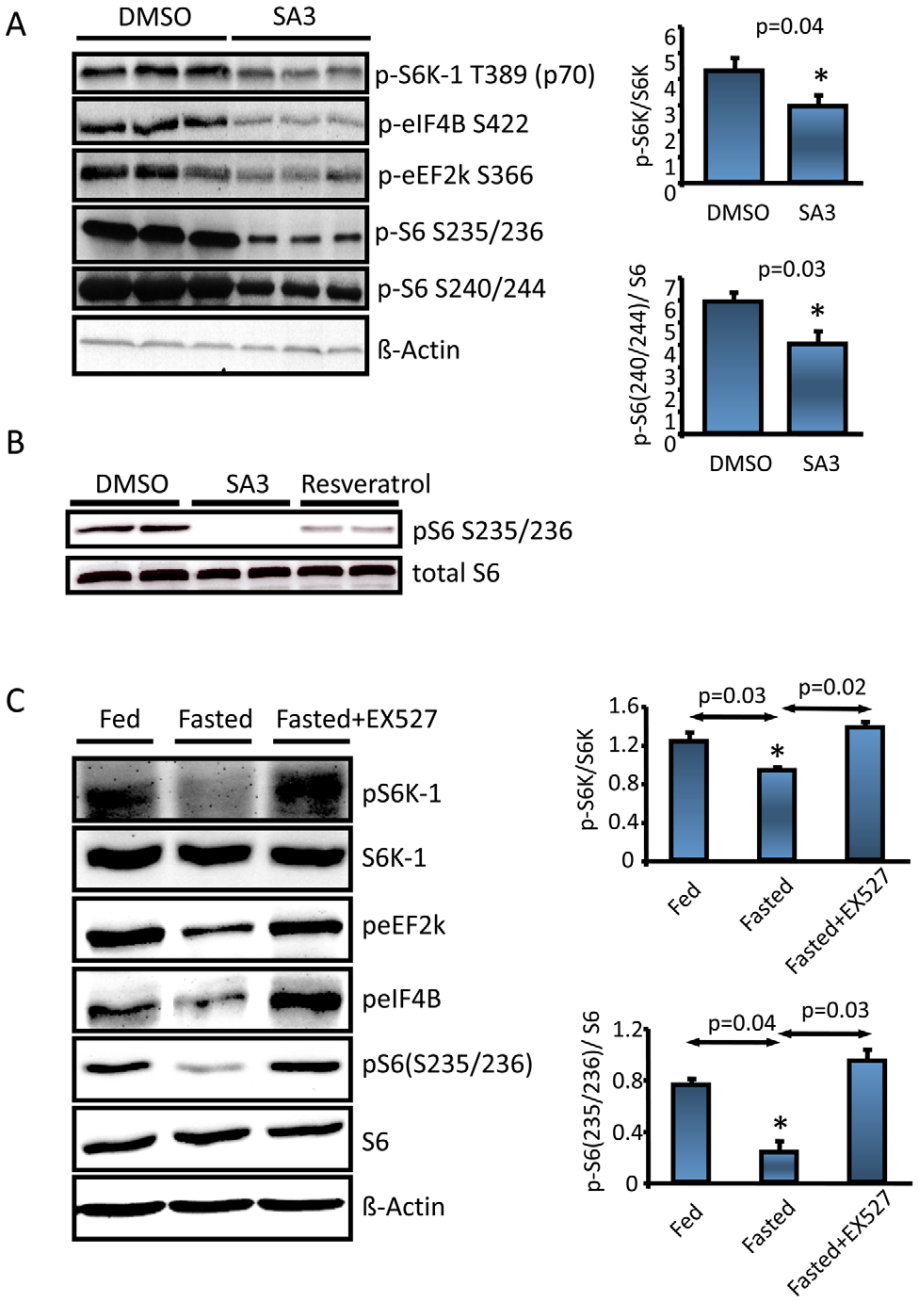
Hypothalamic Sirt1 regulates S6K signaling. (*A*–*B).* N43 cells were serum starved overnight the day before the SA3 or Resveratrol (50 µM each, 6 hours) treatment. 6 hr post treatment, expression level of the proteins was determined by western blotting. (*C*) Inhibition of hypothalamic Sirt1 reverses the fasting induced downregulation of mTOR signaling. 300 g lean, cannulated rats either ad libitum fed (icv infused with DMSO) or fasted (icv infused with DMSO or EX527 once at 40 hr and once 48 hr. Animals were sacrificed 1 hr post last infusion, and a western blot was run from ARC lysates.

It has been recently demonstrated that the hypothalamic mTOR/S6K pathway negatively regulates energy balance under nutritional and hormonal regulation [12], [32], and Sirt1 or its activator resveratrol were suggested to repress S6K signaling [33], [34]. Therefore we asked whether the metabolic action of hypothalamic Sirt1 also involves S6K signaling. Activation of Sirt1 in N43 cells, by either resveratrol or SA3 resulted in a decrease in the phosphorylation of S6K and its downstream substrates (Figure 5A, Figure 5B, Figure S5 top panels), suggesting that Sirt1 inhibits this pathway in the mTOR signaling. Since fasting was reported to modulate hypothalamic S6K activity, we further analyzed whether fasting induced hypothalamic Sirt1 activity was involved in this regulation. As shown in Figure 5C (Figure S5 bottom panels), inhibition of the hypothalamic Sirt1 activity reversed the fasting-induced decrease in S6K signaling.

## Discussion

In this study, we present data supporting the hypothesis that fasting induced hyperphagia requires hypothalamic Sirt1 activity. This conclusion is consistent with the observation that Sirt1 protein levels and activity were increasing in fasted mouse [10] and rat hypothalamus. Several lines of evidence in our study demonstrate that the hypothalamic action of Sirt1 is to regulate food intake and body weight. First, the EX527 treated rats had decreased food intake and body weight gain. These animals' weight gain was less than their pair-fed counterparts suggesting that the decreased in weight gain was not exclusively due to the reduced food intake. Second, Sirt1 showed to regulate the expression of AgRP and POMC. The POMC derived peptide α-MSH decreases food intake, and promotes energy expenditure through melanocortin receptors [35] and activation of HPT axis [21]. AgRP, on the other hand, is a strong antagonist of the melanocortin receptors, and blocks α-MSH action. Blocking the melanocortin receptors consistently abolished the decreased food intake and body weight gain in hypothalamic Sirt1 inhibited animals, showing that melanocortin receptors mediate the effect of Sirt1 on energy balance. Third, inhibition of hypothalamic Sirt1 resulted in increased levels of serum thyroid hormones, which are strong stimulators of basic metabolic rate and thermogenesis.

**Figure 6:**
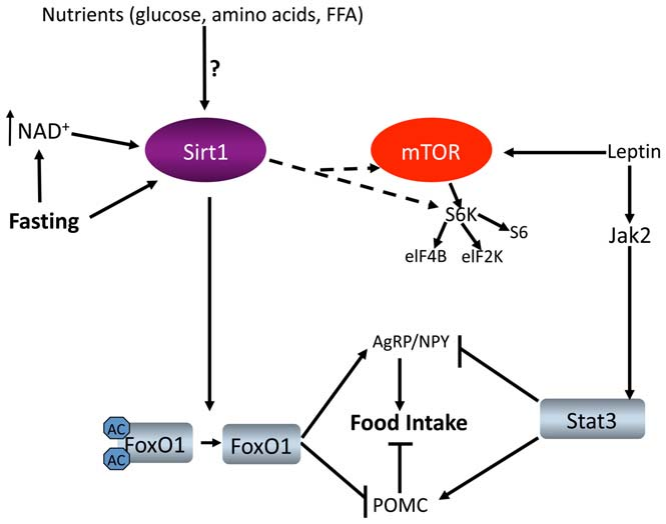
Model depicting Sirt1 action in the hypothalamus. Insulin and leptin activate PI3K, which in turn negatively regulates FoxO1 by phosphorylation. Whether insulin and/or nutrients have direct effects on hypothalamic Sirt1 signaling is unknown. Fasting increases Sirt1 protein level and NAD+ levels, which results in increased Sirt1 deacetylase activity. Sirt1 deacetylates and activates FoxO1, and regulates POMC and Agrp expressions. Hypothalamic Sirt1 action also involves S6K signaling. Whether Sirt1 acts upstream of down-stream of mTOR remains to be established.

In the present work, we studied the acetylation and/or activity of two of the transcriptional regulators of POMC: Stat3 and FoxO1, both of which are shown to be Sirt1 substrates [28], [29]. Stat3 acetylation triggers its dimerization, DNA binding and thus transcriptional regulation [30]. Since Stat3 is a positive regulator of POMC transcription, a potential deacetylation of Stat3 by Sirt1 might render Stat3 inactive, leading to POMC downregulation. On the other hand, FoxO1 is a negative regulator of POMC [26], [27]. Deacetylation of FoxO1 by Sirt1 was shown to stimulate its DNA binding, and transcriptional activity [2], [29], [36]. Under our experimental conditions we could not detect any significant change in the acetylation of ARC Stat3 by fasting, inhibition of Sirt1, or leptin administration, working against the hypothesis that Sirt1 targets Stat3 in the hypothalamus. However, upon fasting, ARC FoxO1 acetylation decreases in a Sirt1 dependent manner (Figure 4B), and Sirt1 cofactor NAD^+^ and Sirt1 protein levels increase (Figure 2), accounting for the increased Sirt1 activity (Figure 4A, B). Also, the fasting induced decrease in POMC expression and increase in AgRP expression were sensitive to Sirt1 enzymatic activity (Figure 3A, B). Therefore we propose that by regulating melanocortin signaling, Sirt1 links nutrient availability to energy homeostasis at the hypothalamic level (Figure 6). This effect is mediated, at least in part, by the fasting induced and Sirt1 mediated deacetylation of FoxO1, such that Sirt1 shifts FoxO1's activity to resist the stress of fasting.

It was recently reported that Sirt1 and AMPK could activate each other [37], [38], [39]. If such an interaction is true in hypothalamic neurons as well, then AMPK might be mediating some of the effects of hypothalamic Sirt1 on energy balance. Randy Seeley's group recently challenged this possibility where they showed that central administration of resveratrol stimulated food intake in rats (Li et al., Resveratrol Regulates Food Intake and Glucose Homeostasis in the CNS, Endocrine Society's 91^st^ Annual Meeting, Washington D.C. [P1-379]). They proposed that resveratrol induced food intake by activating AMPK via Sirt1 activation in the hypothalamus. Therefore, it is very likely that Sirt1 mediated regulation of food intake involves multiple pathways, and includes other metabolic sensors such as AMPK, or mTOR. Our results on the regulation of S6K signaling by Sirt1 are of particular importance. Activation of hypothalamic mTOR or increased expression of S6K in the mediobasal hypothalamus results in decreased feeding [12], [32]; therefore, Sirt1 down-regulating S6K signaling fits our model on the regulation of energy balance by hypothalamic Sirt1. Fasting induced down-regulation of hypothalamic S6K signaling is reversed by leptin as well as by inhibition of Sirt1. The exact molecular mechanism of either regulation is not known yet. Leptin and Sirt1 might be following independent pathways to ultimately regulate S6K signaling. Alternatively, Sirt1 and leptin action might regulate a common mediator of energy balance, such as AMPK [40], which might then activate or inhibit the mTOR signaling according to the specific input. Future research will help to identify the complex regulation of metabolic sensors by hormonal signals for the ultimate regulation of energy homeostasis.

Inactivation of hypothalamic Sirt1 resulting in an anorectic phenotype might seem contradictory to some previous findings on Sirt1. For example, mice whose food was supplemented with resveratrol did not eat more than their control counterparts [41]. However, it is not known whether resveratrol taken orally will effectively reach all arcuate neurons. Resveratrol is also an activator of AMPK [42], which at the central level positively regulates food intake and body weight gain. However, resveratrol fed mice did not show increased food intake or body weight gain [41]. It is therefore very likely that the peripheral effects of resveratrol might compensate for the responses induced at the central level. Recently Ramadori *et. al.* reported that chronic central infusion of resveratrol did not alter food intake or AMPK activity [43]. While the latter result conflicts with previous findings on resveratrol and AMPK [42], our results from central SA3 infusions agrees with Ramadori *et. al.* [43] such that the SA3 induced food intake is a short term response. It is possible that the acute (as reported here) versus chronic actions of hypothalamic Sirt1 might be different. Sirt1 overexpressing mice have been generated and their physiology has been examined. Although one study showed that Sirt1 overexpressing mice ate less than wild type animals [44], two other studies reported these mice being hyperphagic [45], [46]. Whether the observed effects of Sirt1 over expression on food intake involve the hypothalamic Sirt1 action remains to be determined. In summary, our study establishes a physiological role for hypothalamic Sirt1. We propose that Sirt1 at the central level senses the nutritional status of the body and regulates hypothalamic central melanocortin signaling and S6K pathway to govern food intake and body weight (Figure 6). Given the role of Sirt1 in several peripheral tissues and hypothalamus, therapies centered on Sirt1 present promising results for the treatment of metabolic diseases such as obesity.

## Materials and Methods

### Reagents and Antibodies

Resveratrol and Trichostatin A were purchased from Calbiochem (La Jolla, CA). EX-527 (6-Chloro-2,3,4,9-tetrahydro-1H-carbazole-1-carboxamide) was from Alexis Biochemicals (San Diego, CA). Sirt1 Activator 3 (SA3) was from Cayman Chemical (Ann Arbor, MI). Sirt1 antibody was from Upstate (Anti-Sir2, Lake Placid, NY). ACTH/CLIP antibody used in western blots to detect POMC or 21 kDa ACTH precursor was from Santa Cruz (Santa Cruz, CA). anti-aMSH antibody and the anti-ACTH antibody (used in RIA) were developed in our laboratory and fully described earlier [24]. Other reagents were mentioned throughout the text.

### Cell Culture

The mouse corticotropic tumoral cell line AtT20 was cultured in DMEM (Invitrogen, Carlsbad, California) with 10% FBS. The cells were serum starved overnight the day before resveratrol (5 or 50 µM) treatments. For fold stimulation analysis, cells were incubated with serum free media for 5 hours, and media were collected. Then equal amounts of resveratrol or SA3 were added to the same dishes in a serum free media. The media were again collected after 5 hours, speed vacuumed overnight, and analyzed for ACTH and TRH peptides by radioimmunoassay (RIA). The fold stimulation is simply represented by the ratio of the amount of peptide in the second media over the amount in the first media from the same dish. To knock-down Sirt1 or FoxO1 in AtT20s, specific siRNAs (Invitrogen) were mixed with Lipofectamine 2000 (Invitrogen, Carlsbad, California) diluted in DMEM. The siRNA-Lipofecetamine mixture was added onto the cells. The medium was changed 5 hours post-transfection. The resveratrol treatment was done 60 hours post transfection. Cells were lysed in RIPA buffer (50 mM Tris-HCl, pH: 7.4, 150 mM NaCl, 100 mM NaF, 1 mM EDTA, 0.5% Sodium Deoxycholate, 0.1% SDS, 1% NP-40) supplemented with protease and phosphatase inhibitor cocktails. Sirt1, POMC, FoxO1, and β-Actin levels were analyzed by western blot. The hypothalamic cultures were prepared as previously described [21], [25]. Briefly, the diencephalons were excised from rat fetuses on day 17 of gestation. After enzymatic treatment with trypsin and DNase I to dissociate the diencephalons into single cells, cells were plated onto six-well dishes coated with poly-D-lysine. To prevent glial cell growth, cytosine arabinose (Sigma-Aldrich, St Louis, MO) was added on day 4 of the culture. The culture was kept for two weeks before any treatment was done, with half of the culture media being replaced with fresh media every fourth day.

### Animal Studies and ICV Infusions

Male Sprague Dawley rats were obtained from Charles River Laboratories, Inc. (Wilmington, MA). The Institutional Animal Care and Use Committee of Rhode Island Hospital/Brown University approved the experimental protocols and euthanasia procedures. Animals had free access to water all the time, and fed *ad libitum* a regular diet (Purina Lab Chow #5001, Ralston Purina Corp, St. Louis, MO) except when indicated in the experiments. Animals were kept at a 12 hours dark/light cycle, light being off between 18:00 and 06:00. For the intra-cerebro-ventricular (ICV) infusion experiments, 275–300 gr rats were stereotaxically implanted with an intra-cerebro-ventricular (ICV) cannula (Plastic One, VA) 10 days before the experiment. The placement coordinates for the lateral ventricle were: anteroposterior:−0.8 mm, lateral:−2.0 mm, and ventral:−3.6 mm from bregma. The correct placement of the cannulas was verified by measurement of water intake in response to ICV injection of Angiotensin II (40 ng/rat). Rats were sacrificed by decapitation, brains were immediately removed, and ARCs were excised between the rostral and caudal limits of the ME and 0.5 mm to each lateral side of the ME. The depth of each section isolated was 1 mm thick, in parallel to the base of the hypothalamus. ARCs were processed accordingly. Leptin was administered when indicated intra-peritoneally at a dose of 1 µg per gram rat. Animals were sacrificed 3 hours afterward.

### Food Intake Study

Individually caged cannulated fasted rats with body weight and age-matched were treated as follow:

EX-527 (5 µg) in 2 µL DMSO + 4 µL artificial cerebrospinal fluid (aCSF),

SA3 (3 µg) in 2 µL DMSO + 4 µL artificial cerebrospinal fluid (aCSF),

SA3 (3 µg) + EX-527 (5 µg) in 2 µL DMSO + 4 µL artificial cerebrospinal fluid

1 µg SHU9119 (in 1 µL aCSF) + 2 µL DMSO + 3 µL aCSF,

1 µg SHU9119 + 5 µg EX-527 in 2 µL DMSO + 3 µL aCSF,

2 µL DMSO + 4 µL aCSF

Treatments were performed once in the morning (6 µL at 40 hr fasting for Figure 1A, and at 16 hr for Figure S1D), and once one hour before the dark cycle (6 µL at 48 hr fasting for Figure 1A, and at 24 hr for Figure S1D). The food was returned to the cages right before dark cycle. The body weight was measured at the time of second ICV injection (48 hr fasting for Figure 1A, and at 24 hr for Figure S1D) and after 16 hours. Food intake was measured in the morning when animals were weighed. The average amount of food eaten by EX-527 group was given to the pair fed group treated with DMSO+aCSF solution. The pair-fed group animals consumed all the food overnight. Of note, DMSO alone did not change food intake compared to aCSF alone (data not shown). For the SA3 ICV experiments, 36 hour fasted and cannulated rats were re-fed for 3 hours. At the end of re-feeding (during the light phase), 6 µg SA3 (in DMSO) or just DMSO (4 µL) was infused into the lateral ventricle. The food intake was measured at the indicated time points in Figure 1A. The experiment was repeated in O/N fasted + 3 hr re-fed rats, where the results were the same as the presented results in Figure 1A right panel (data not shown). For the knock-down experiments, rats under anesthesia were placed double cannulas into the ARC. The placement coordinates for the ARC were: anteroposterior:−2.6 mm, lateral:−0.5 mm, and ventral:−9.0 mm. Injector cannulas were designed to have a 0.5 mm projection from the end of the cannula, meaning the actual ventral coordinate of injection was −9.5 mm. siRNAs to be injected were prepared according to the in vivo siRNA transfection protocol for brain delivery from PolyPlus Transfection (New York, NY) [47], [48], [49], [50]. Briefly, 5 µL DOPE (1,2-Dioleoyl-sn-glycero-3-phosphoethanolamine, Sigma-Aldrich, St Louis, MO) dissolved in chloroform and ethanol (final concentration 80 mM) was mixed with 20 µL jetSI™ (10 mM from PolyPlus Transfection, New York, NY) to form Solution A. Solution A was diluted first with ethanol (Solution B) and then with 25% glucose in order to obtain the solution C with a final glucose concentration of 5%. 5,6 µg negative control siRNA (Qiagen, Valencia, CA) or siRNA targeting rat Sirt1 (Dharmacon, Inc., Lafayette, CO) were diluted into a final glucose concentration of 5%. Solution C was added into the siRNA solution in a 1∶1 (v/v) ratio. After 30 min incubation at room temperature and in less than one hour, 1 µL of the siRNA complexes were injected into each side of the third ventricle into the ARC of rats under anesthesia. The animals were individually caged, and food intake and body weight were measured every day. 2, 5 or 10 days after the injection some animals were sacrificed and the ARC Sirt1 protein levels were determined by western blotting. Knock down efficiency was similar at the days analyzed. FoxO1 siRNAs were prepared similarly. 4 µL was injected ICV at 08:00hr and 16:00hr every day for 4 days. The rats were fasted the last 24 hours for the EX527 infusions, which were done once at the beginning of fasting and another time in the following morning. Rats were sacrificed after 25 hours of fasting.

### Western Blotting

Ad libitum fed or fasted rats were sacrificed by decapitation and the brains were immediately removed. ARC and PVN were excised, and lysed in RIPA buffer containing a mixture of protease and phosphatase inhibitors (Sigma-Aldrich, St Louis, MO) supplemented with 10 mM Nicotinamide, 10 µM Trichostatin A, and 10 µM EX527. Protein quantification was done by Bradford assay. The Precision Plus Protein standards were used as molecular mass markers (Bio-Rad Laboratories, Richmond, CA). After separation on SDS PAGE, the proteins were transferred to PVDF membranes, and blocked with 5% dry milk (5% BSA was used for the phosphorylated proteins) in TBS with 0.1% Tween-20 (TBS/T). For Sirt1 analysis, the membranes were cut into two from 75 kDa marker line and lower parts were incubated with mouse anti-beta-Actin antibody (Santa Cruz, 1∶10000), top parts were incubated with rabbit anti-Sirt1 antibody (Upstate, 1∶1500). Figure S1E serves as a control that this antibody can be used to detect rat ARC Sirt1 by western blotting. For detection of acetylated p53 (1∶500, Cell Signaling), acetylated FoxO1 (1∶200, Santa Cruz), acetylated Stat3 (1∶500, Cell Signaling), and phospho-Stat3 (1∶1000) the blots were incubated with the modification specific antibodies, and developed, followed by β-mercaptoethanol stripping, and incubation with the total p53 (1∶200, Santa Cruz), FoxO1 (1∶1000, Cell Signaling), Stat3 (1∶200, Santa Cruz) antibodies for evaluation of equal protein loading. HRP conjugated goat anti-mouse, goat anti-rabbit, and rabbit anti-goat secondary antibodies (Bio-Rad Laboratories, Richmond, CA) were used for detection of the protein bands. All primary antibody incubations were done at 4°C overnight, except for the acetylated-Stat3 antibody, where the incubation was for 48 hours at 4°C. Secondary antibody incubations were done at room temperature in 5% non-fat dry milk in TBS/T for 1 hour (2 hour for the acetylated Stat3 antibody). Band intensities were quantified by NIH Image J software.

### Adenoviral Infection

Control shRNA and Sirt1 shRNA adenoviruses were kindly provided by Dr. Pere Puigserver (Dana-Farber Cancer Institute, Department of Cell Biology, Harvard Medical School, MA, USA). For the infection of hypothalamic cultures 1,5×10^7^ infectious particles (per well) were added into the media of six well dishes at day 14 of the culture. 4–5 days later, cells were added fresh media with 0.1% BSA and 0.1% FBS. The media was collected 6 hours later for the α-MSH RIA. Cells were lysed in RIPA buffer; protein content was determined by Bradford assay. Ten µg of protein was run on SDS-PAGE to assess the Sirt1 knock-down efficiency.

### Double Immuno-Histochemistry (IHC) for Sirt1 and ACTH

Rats were anesthetized with pentabarbitol and systemically perfused with 4% paraformaldehyde solution. Brains were removed and further fixed for 2 hours, and then kept in 20% sucrose solution at 4°C overnight. Frozen brains were cut in 25-mm-thick coronal sections on a sliding cryostat. Tissue samples were pretreated with 1% NaOH and 1% H_2_O_2_ in H_2_O for 20 minutes, 0.3% glycine for 10 minutes, and 0.03% SDS for 10 minutes. After that, sections were blocked for 1 hour with 3% normal goat serum in PBS/0.25% TritonX-100/0.2% sodium azide. The anti-Sirt1 antibody in blocking solution was then added (1∶1,000) and incubated overnight at 4°C. On the next day, sections were washed, incubated with biotinylated secondary goat anti-rabbit antibody for 2 hours (1∶1,000), and then treated with avidin biotin complex solution for 1 hour. Finally, the signal was developed with diaminobenzidine solution, giving a brown precipitate. Consecutively, IHC for ACTH was performed by incubation overnight at 4°C with the primary antibody (1∶2,000) in blocking solution. On the next day, sections were washed, incubated with a fluorescent secondary goat anti-rabbit Alexa 596 (red) antibody for 1 hour. Results were visualized using either fluorescence (ACTH) or bright-field light (Sirt1) sources. Fluorescent images (12 bit) and DAB images (24 bit) were acquired with a Nikon E800 microscope (Nikon Inc. Melville NY) and a Spot II digital camera (Diagnostic Instruments, Sterling Heights MI). Using ImageJ (NIH, Springfield, VA) and Adobe Photoshop (Adobe, San Jose, CA) fluorescence and bright-field photographs were combined using RGB channels to visualize double-labeled cells.

### Radioimmunoassay Analysis (RIA)

The assays used for α-MSH and ACTH-derived peptides were previously developed in our laboratory [24]. Primary antibodies were also developed in our laboratory. Each peptide was iodinated with 125I using the chloramine T oxidation-reduction method followed by HPLC separation, and the purified peptide was used as the tracer. The α-MSH RIA assay was performed in 0.5 ml of phosphate buffer (pH 7.4)/500 mg/l sodium azide-2.5 g/l BSA, with primary anti-α-MSH antiserum (1∶20,000) and 5,000 cpm of 125I-desacetyl α-MSH tracer. The α-MSH assay used under these experimental conditions detected 100% of acetyl and desacetyl α-MSH of the amidated forms. The α-MSH assay has no cross-reactivity with CLIP [24]. The ACTH RIA assay was performed in 0.5 ml of RIA buffer containing anti-ACTH antiserum (1∶30,000) and 5,000 cpm of 125I-ACTH tracer. The ACTH assay used under these experimental conditions can detect 100% of CLIP and ACTH forms; however, this assay does not cross-react with any form of α-MSH. The RIA kits from MP Biomedicals Diagnostic Division (Orangeburg, NY) were used to measure serum T_3_ and T_4_ levels. Similar results were obtained when the experiment was done on 24 hour-fasted rats (data not shown) as opposed to 48 hour-fasted rats (Figure 1D). For the detection of ARC α-MSH, fed or fasted extracted rat ARCs were boiled in 2N acetic acid supplemented with protease inhibitors, and then sonicated. After centrifugation, protein/peptide concentration was determined by Bradford assay. Equal amounts of total protein were speed vacuumed for α-MSH RIA.

### Real Time RT-PCR

Cannulated rats fasted for 25 hours were infused with DMSO (control group) or EX-527 once at the beginning of the fasting period and a second time in the following morning (16 hr of fasting). Rats were sacrificed after 25 hours of fasting. Brains were immediately removed, and ARCs were excised. Total RNA (1 µg) isolated (Trizol Reagent, Invitrogen) from fresh arcuate nucleus tissue was converted to cDNA and used to screen expression levels of Sirt1, POMC, AgRP, and tubulin (internal control). Reactions were amplified in an ABI Prism 7500 FAST sequence detector (Applied Biosystems) and acquired data were analyzed using the ΔΔCt method to determine the expression level of each transcript normalized to the expression level of the housekeeping gene (tubulin). RNA from AtT20s was isolated with Trizol and the described protocol is followed. The primer sequences used for real time PCR are available upon request.

### NAD^+^- NADH Measurements

NAD^+^-NADH measurements were done using BioVision (Mountain View, CA) NAD+/NADH Quantification kit according to the manufactures instructions. Briefly, ARCs were sonicated in the NADH/NAD extraction buffer. Samples were filtered through 10kDa molecular weight cut off filters (Millipore). Half of the eluate was used to determine NAD total (NADt = NADH + NAD^+^). The other half was heated to 60°C for 30 minute, and used for NADH measurements. NAD^+^ was simply calculated by NAD^+^ = NADt-NADH. The reactions were prepared in 96 well dishes, and read at OD_450_. The concentrations were determined after applying the reading to the linear part of the standard curve generated by the NADH standard supplied.

### FKHR Transcription Factor Assay

N43 or hypothalamic nuclear isolation was done using Nuclear Extract Kit (Active Motif, CA). Nuclear lysates were used without freeze-thaw cycles for FoxO1 activity assay using FKHR Transcription Factor Assay kit according to manufacturer's instructions. Briefly, nuclear lysates were added into the provided 96-well plate. After 1 hr incubation at room temperature, the wells were washed, and subsequently incubated with the primary and, HRP-conjugated secondary antibodies. The color development was carried out for 6–7 minutes. The OD450 was normalized to the total nuclear lysate added per well. Each sample was run in triplicate per assay. The detected signal's specificity was confirmed by addition of wt vs. mutated oligonucleotides provided with the kit, such that the signals were reversed by the addition of wt but not mutant oligonucleotides (data not shown).

### Statistical Analysis

Results were expressed as average ± SEM, (n is given in the Figure legends). Statistical significance of differences was calculated with Student's t test.

## Supporting Information

Figure S1 A-G. Sirt1 is expressed in the rat hypothalamus, and modulation of its activity or expression alters food intake. (A) Coronal brain sections of an ad libitum fed rat subjected to IHC (immunohistochemical staining) using an antibody against Sirt1. Sirt1 is expressed in the nuclei of the hypothalamus involved in energy homeostasis: (Left) paraventricular nucleus (PVN), (Middle) arcuate nucleus (ARC) and Median Eminence (ME), (Right) ARC in higher magnification. 3 rats were examined for their hypothalamic Sirt1 expression pattern. (3V: Third Ventricle, FX: Fornix, OPT: Optic Tract)). (B) (Left) Analysis of Sirt1 expression by western blotting. Sirt1 is present in the ARC, PVN, primary hypothalamic cultures obtained from rat embryo diencephalons (Hyps), and the AtT20 cell line. An equal amount of protein (20 µg) was run on SDS-PAGE for the western blot. (Right) Whole hypothalamic lysate (20 µg) was run on an 8% gel, and a western for Sirt1 was done. (C) Light micrographs showing the immunohistochemical (DAB staining) distribution of Sirt1 in hippocampus (left) or cortex (right). (D) (Left and middle panels) Food intake and body weight gain of 24 hr fasted rats, which were DMSO or EX527 infused at 16 hr and 24 hr of fasting. Food intake and body weight gain were measured 24 hours after the last infusion, at which point the food was added into the cages. (Right panel) Hypothalamic acetylated-p53 level decreases after central infusion of SA3. (E) (Left) Knock-down of Sirt1 expression by intra-ARC siRNA infusions. Top panel shows western blots for Sirt1 using the ARC samples obtained 48 hr after siRNA infusion. β-actin is used as the loading control. Bottom graph shows the quantification of the western blot on top. ARC Sirt1 protein levels decreases around 40% upon siRNA infusion. Values are normalized to β-actin levels. n = 4 per group. (Right) ARC Sirt1 protein levels 5 days post-injection. At 10 days post-injection (data not shown), Sirt1 protein levels were very similar to 5 days post-injection. (F) Sirt1 staining pattern changes upon Sirt1 knock-down only in the ARC. Sirt1 IHC after infusion of (-) ctrl siRNA (right side of 3V) or Sirt1 siRNA (left side of 3V) into the ARC. Top panel ARC, bottom PVN. (G) Quantification of the Sirt1 positive nuclei in either side of the ARC from (B). 4 animals per group and 6 sections per animal were analyzed. *, p<0.05 vs. (-) Ctrl siRNA. Values are the mean ± SEM. 3V, third ventricle. *, p<0.05 vs. Ctrl.
(10.28 MB TIF)

Figure S2 A-C. Sirt1 is expressed in POMC neurons. (A) IHC with ACTH antibody to stain POMC neurons in the ARC. (B) Immunohistochemical staining for Sirt1 and POMC in the ARC. Top row shows lower magnification IHC for Sirt1 and POMC in coronal rat brain sections of ARC. All POMC neurons express Sirt1. Bottom row shows higher magnification images of the top panels. (C) α-MSH level decreases in the 48-hour fasted rat ARC compared to the fed animals. n = 5. Values are the mean ± SEM. *, p<0.05 vs. fed.
(10.04 MB TIF)

Figure S3 A-D. Sirt1 regulates POMC expression. (A) Post-translational processing of POMC. POMC is first cleaved into two giving rise to the 21 kDa ACTH precursor, which is then processed into ACTH and ultimately into α-MSH in the ARC and hypothalamic cultures. AtT20 cells process POMC up to ACTH since they lack the enzyme PC2, which cuts ACTH to produce α-MSH. (B) Knock-down of Sirt1 in rat primary hypothalamic cultures (Hyps). Sirt1 shRNA Adenovirus or the negative control shRNA adenovirus infected hypothalamic cultures were analyzed -by western blotting- for the Sirt1 expression. n = 4. (C) α-MSH secreted from Hyps in (B) is increased upon Sirt1 knock-down. (D) Resveratrol (50 µM) or SA3 (40 µM) treatment decreases ACTH released into the culture media. Media were collected from the cells in (Figure 3E), and analyzed by RIA. Amount of TRH secreted (negative control, data not shown) from preproTRH transfected AtT20s is not affected by the resveratrol or SA3 treatment (n = 3, per condition. The experiment was done twice).
(8.92 MB TIF)

Figure S4 A-C. Inhibition of hypothalamic Sirt1 activity, and icv infusion of FoxO1 specific siRNAs. (A) FoxO1 western using total rat hypothalamic lysate. (B) Elevation of the acetylated p53 levels in the hypothalamus of rats icv treated with the Sirt1 inhibitor EX527. (C) icv infusion of FoxO1 specific siRNAs results in decreased FoxO1 protein levels.
(10.31 MB TIF)

Figure S5 Hypothalamic Sirt1 regulates S6K signaling. Densitometry of the western blots presented in Figure 5A (top three graph), and Figure 5C (bottom two graphs).
(9.47 MB TIF)
