# Supplementary figures and images for: Hypothalamic Sirt1 Regulates Food Intake in a Rodent Model System
Source: PLoS One. 2009 Dec 15;4(12):e8322. doi: 10.1371/journal.pone.0008322 (PMC2790615; doi:10.1371/journal.pone.0008322)

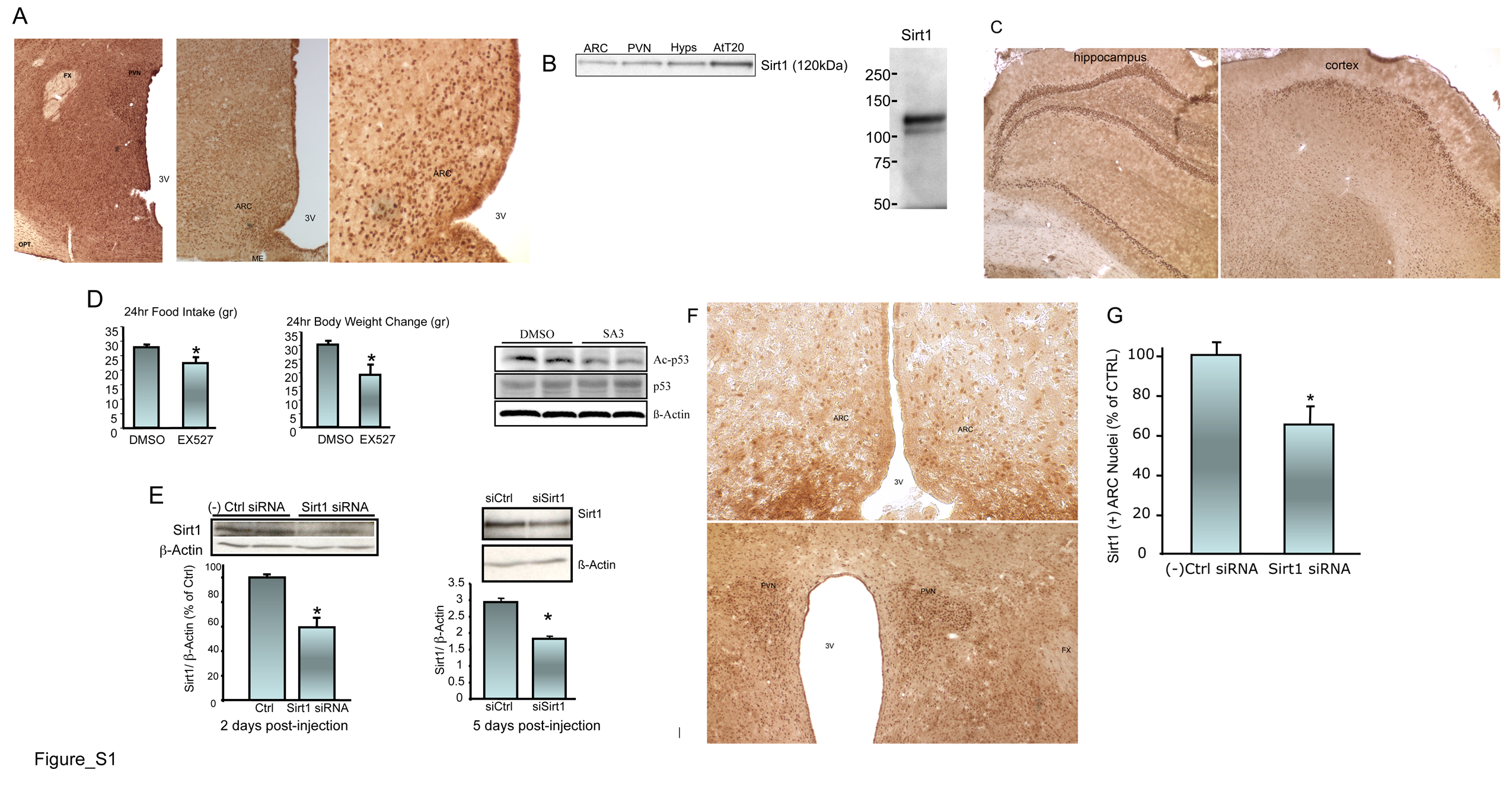

Supplement: Figure S1 — A-G. Sirt1 is expressed in the rat hypothalamus, and modulation of its activity or expression alters food intake. (A) Coronal brain sections of an ad libitum fed rat subjected to IHC (immunohistochemical staining) using an antibody against Sirt1. Sirt1 is expressed in the nuclei of the hypothalamus involved in energy homeostasis: (Left) paraventricular nucleus (PVN), (Middle) arcuate nucleus (ARC) and Median Eminence (ME), (Right) ARC in higher magnification. 3 rats were examined for their hypothalamic Sirt1 expression pattern. (3V: Third Ventricle, FX: Fornix, OPT: Optic Tract)). (B) (Left) Analysis of Sirt1 expression by western blotting. Sirt1 is present in the ARC, PVN, primary hypothalamic cultures obtained from rat embryo diencephalons (Hyps), and the AtT20 cell line. An equal amount of protein (20 µg) was run on SDS-PAGE for the western blot. (Right) Whole hypothalamic lysate (20 µg) was run on an 8% gel, and a western for Sirt1 was done. (C) Light micrographs showing the immunohistochemical (DAB staining) distribution of Sirt1 in hippocampus (left) or cortex (right). (D) (Left and middle panels) Food intake and body weight gain of 24 hr fasted rats, which were DMSO or EX527 infused at 16 hr and 24 hr of fasting. Food intake and body weight gain were measured 24 hours after the last infusion, at which point the food was added into the cages. (Right panel) Hypothalamic acetylated-p53 level decreases after central infusion of SA3. (E) (Left) Knock-down of Sirt1 expression by intra-ARC siRNA infusions. Top panel shows western blots for Sirt1 using the ARC samples obtained 48 hr after siRNA infusion. β-actin is used as the loading control. Bottom graph shows the quantification of the western blot on top. ARC Sirt1 protein levels decreases around 40% upon siRNA infusion. Values are normalized to β-actin levels. n = 4 per group. (Right) ARC Sirt1 protein levels 5 days post-injection. At 10 days post-injection (data not shown), Sirt1 protein levels were very similar [file pone.0008322.s001.tif]

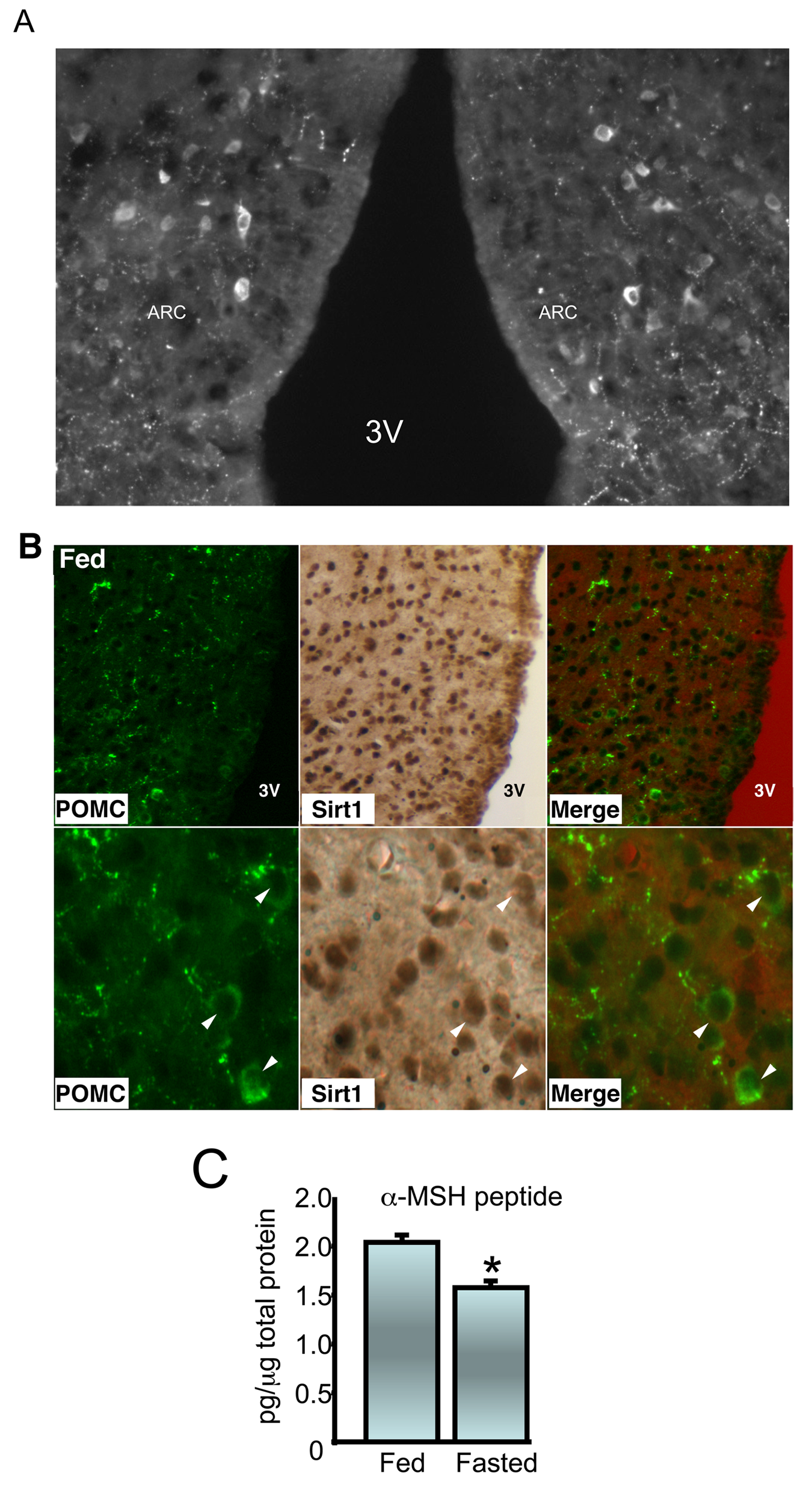

Supplement: Figure S2 — A-C. Sirt1 is expressed in POMC neurons. (A) IHC with ACTH antibody to stain POMC neurons in the ARC. (B) Immunohistochemical staining for Sirt1 and POMC in the ARC. Top row shows lower magnification IHC for Sirt1 and POMC in coronal rat brain sections of ARC. All POMC neurons express Sirt1. Bottom row shows higher magnification images of the top panels. (C) α-MSH level decreases in the 48-hour fasted rat ARC compared to the fed animals. n = 5. Values are the mean ± SEM. *, p<0.05 vs. fed. (10.04 MB TIF) [file pone.0008322.s002.tif]

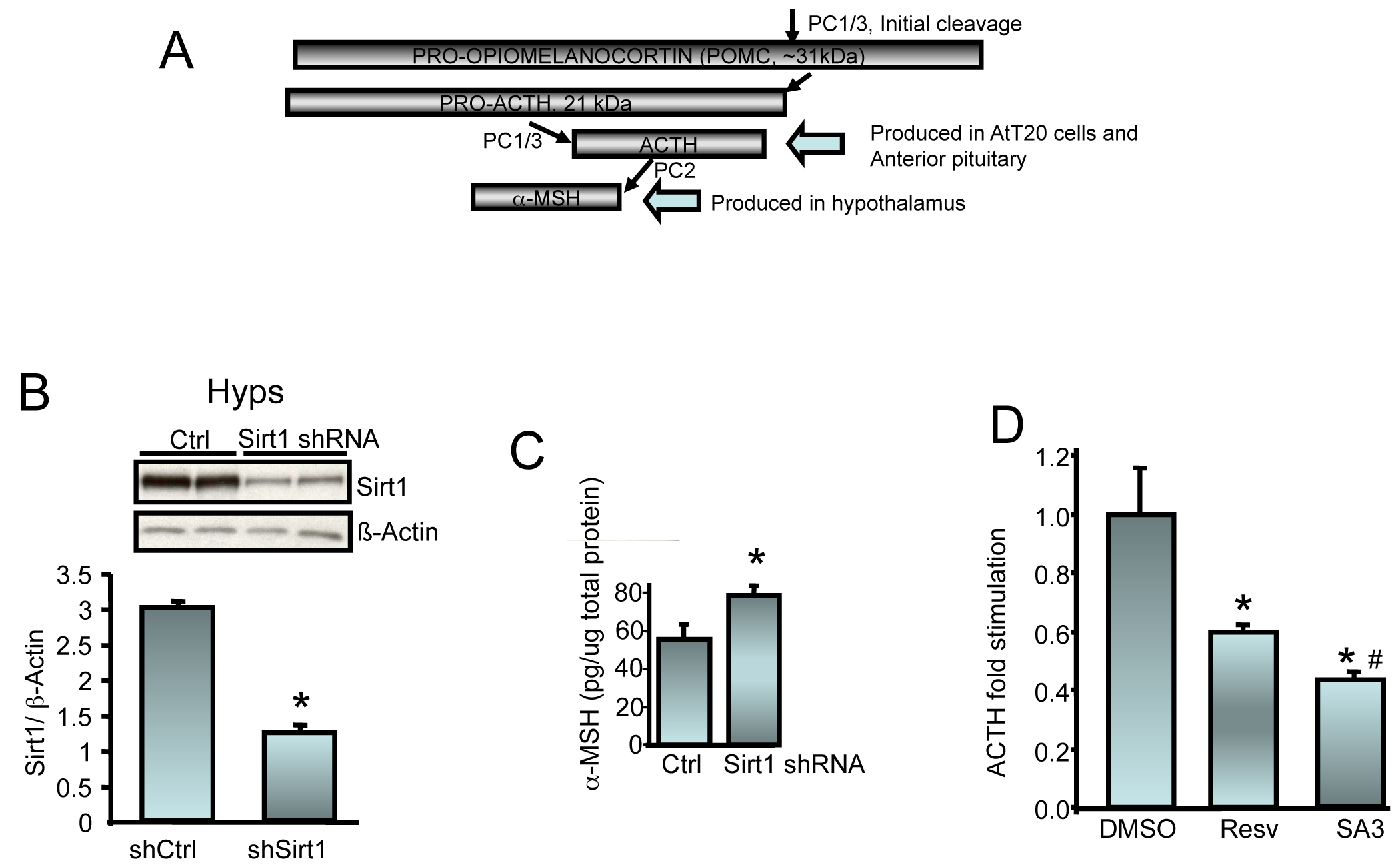

Supplement: Figure S3 — A-D. Sirt1 regulates POMC expression. (A) Post-translational processing of POMC. POMC is first cleaved into two giving rise to the 21 kDa ACTH precursor, which is then processed into ACTH and ultimately into α-MSH in the ARC and hypothalamic cultures. AtT20 cells process POMC up to ACTH since they lack the enzyme PC2, which cuts ACTH to produce α-MSH. (B) Knock-down of Sirt1 in rat primary hypothalamic cultures (Hyps). Sirt1 shRNA Adenovirus or the negative control shRNA adenovirus infected hypothalamic cultures were analyzed -by western blotting- for the Sirt1 expression. n = 4. (C) α-MSH secreted from Hyps in (B) is increased upon Sirt1 knock-down. (D) Resveratrol (50 µM) or SA3 (40 µM) treatment decreases ACTH released into the culture media. Media were collected from the cells in (Figure 3E), and analyzed by RIA. Amount of TRH secreted (negative control, data not shown) from preproTRH transfected AtT20s is not affected by the resveratrol or SA3 treatment (n = 3, per condition. The experiment was done twice). (8.92 MB TIF) [file pone.0008322.s003.tif]

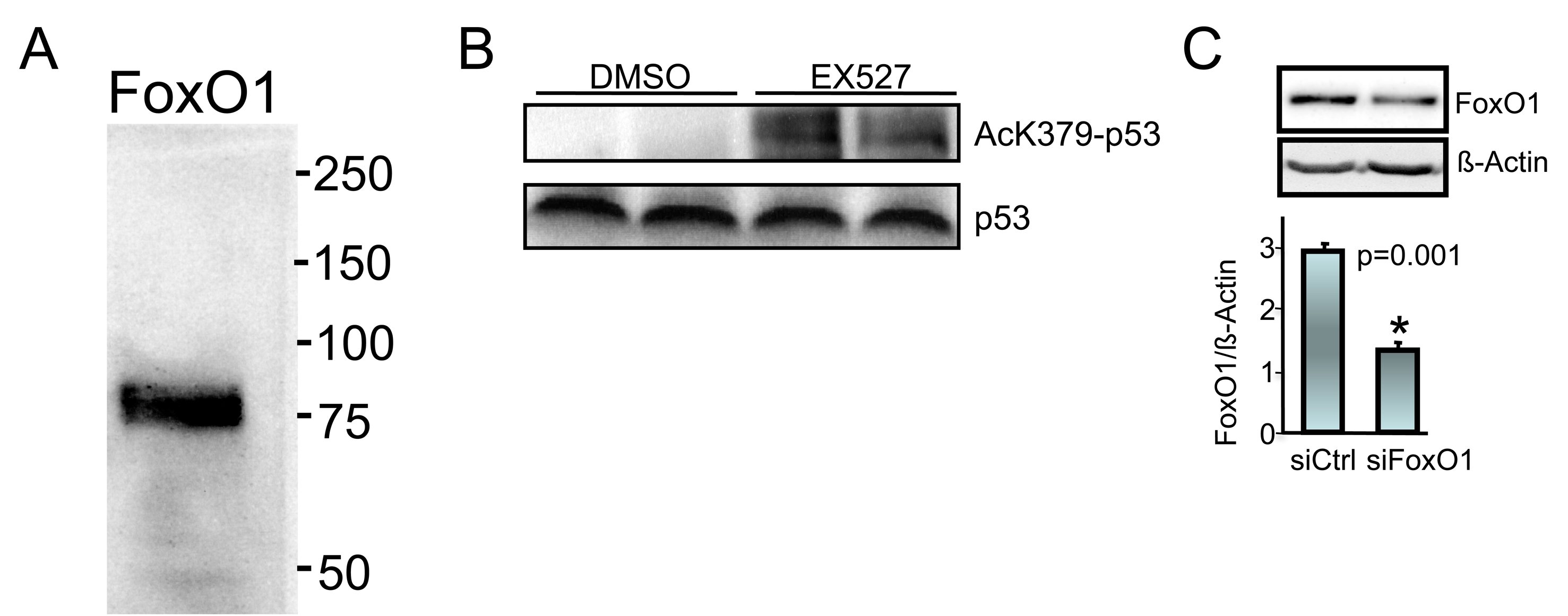

Supplement: Figure S4 — A-C. Inhibition of hypothalamic Sirt1 activity, and icv infusion of FoxO1 specific siRNAs. (A) FoxO1 western using total rat hypothalamic lysate. (B) Elevation of the acetylated p53 levels in the hypothalamus of rats icv treated with the Sirt1 inhibitor EX527. (C) icv infusion of FoxO1 specific siRNAs results in decreased FoxO1 protein levels. (10.31 MB TIF) [file pone.0008322.s004.tif]

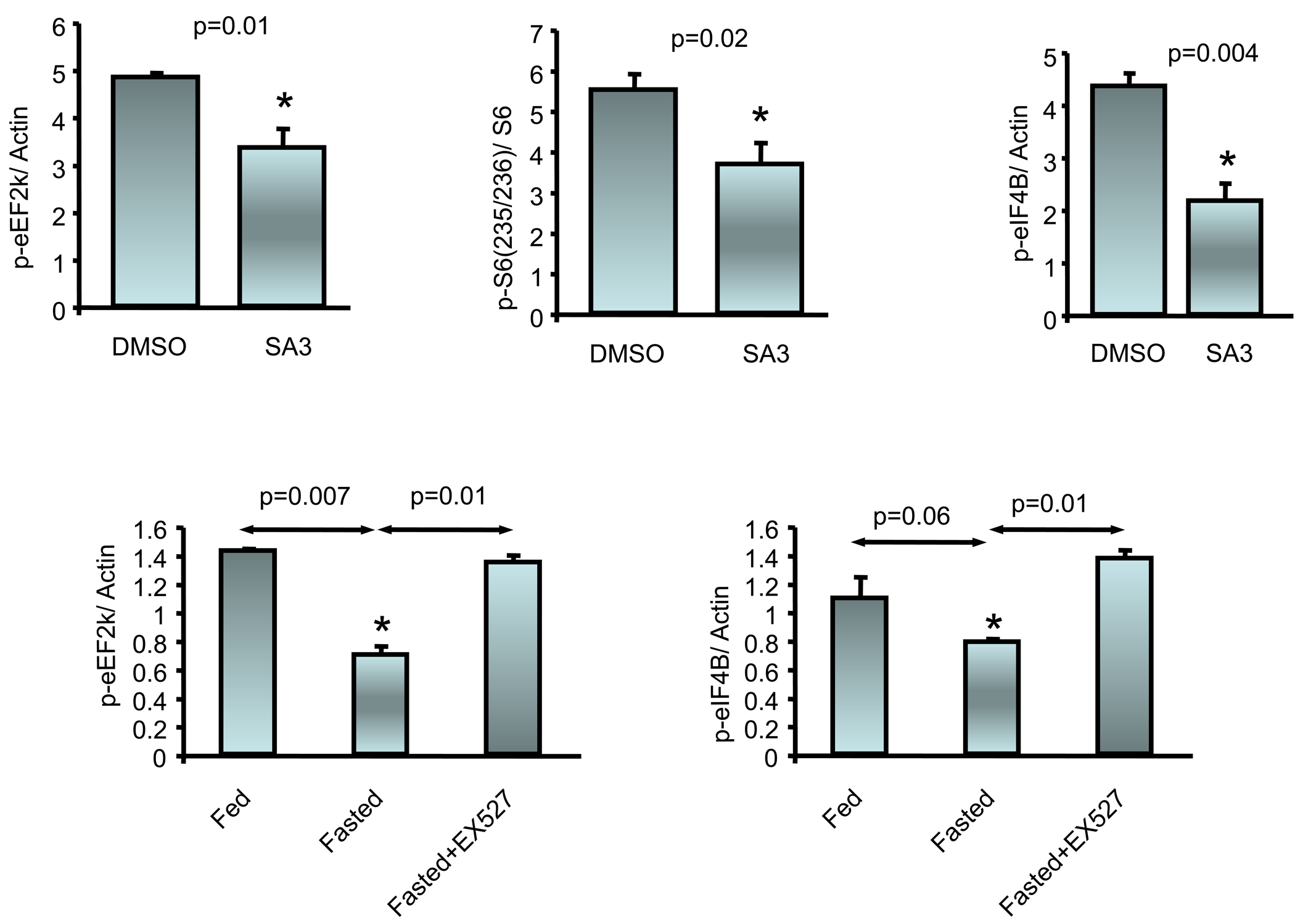

Supplement: Figure S5 — Hypothalamic Sirt1 regulates S6K signaling. Densitometry of the western blots presented in Figure 5A (top three graph), and Figure 5C (bottom two graphs). (9.47 MB TIF) [file pone.0008322.s005.tif]
